# Supplementary material for: Immunogenicity and Safety of Homologous and Heterologous Prime–Boost Immunization with COVID-19 Vaccine: Systematic Review and Meta-Analysis
Source: Vaccines (Basel). 2022 May 18;10(5):798. doi: 10.3390/vaccines10050798 (PMC9142990; doi:10.3390/vaccines10050798)
Supplement: Supplementary file 1 [file vaccines-10-00798-s001.zip › vaccines-1703563-sup-revised.pdf]

Immunogenicity and Safety of Homologous and Heterologous Prime-Boost Immunization with COVID-19 Vaccine: Systematic Review and Meta-Analysis

Haoyue Cheng [1,2], Zhicheng Peng [1,2], Shuting Si [1,2], Xialidan Alifu [1,2], Haibo Zhou [1,2], Peihan Chi [1,2], Yan Zhuang [1,2], Minjia Mo [1,2] and Yunxian Yu [1,2,*]

1. Department of Public Health and Department of Anesthesiology, the Second Affiliated Hospital of Zhejiang University School of Medicine, Hangzhou, China
2. Department of Epidemiology & Health Statistics, School of Public Health, School of Medicine, Zhejiang University, Hangzhou, China

* Correspondence: yunxianyu@zju.edu.cn

Table S1. Literature search terms used for PubMed. The final search strategy applied to conduct this meta-analysis is reported at step #22.

#	**Search strategy**
1	corona virus OR coronavirus
2	coronavirinae
3	coronaviridae
4	betacoronavirus OR beta coronavirus
5	COVID19 OR COVID 19 OR COVID2019 OR COVID 2019
6	nCoV
7	"CoV 2" OR CoV2
8	2019nCoV OR 2019 nCoV
9	severe acute respiratory syndrome coronavirus 2
10	SARS CoV 2 OR sarscov2
11	#1 OR #2 OR #3 OR #4 OR #5 OR #6 OR #7 OR #8 OR #9 OR #10
12	vaccin*[tiab]
13	immuniz*[tiab]
14	inocul*[tiab]
15	#12 OR #13 OR #14
16	#11 AND #15 (29406)
17	"COVID19 vaccin*" OR "anti-sars-cov-2 agent'" OR anti-sarscov2 agent OR "sars-cov-2 vaccine"
18	#16 OR #17 (29514)
19	boost*[tiab] OR third[tiab]
20	#18 AND #19 (2163)
21	Humans[Mesh] OR human*[tiab] OR volunteer*[tiab] OR participant*[tiab] OR subject*[tiab] OR people[tiab]
22	#20 AND #21 (1577)

Table S2. Literature search terms used for the Cochrane Library. The final search strategy applied to conduct this meta-analysis is reported at step #26.

#	**Search strategy**
1	MeSH descriptor: [Coronavirus] explode all trees
2	MeSH descriptor: [Coronavirus 2, SARS] explode all trees
3	MeSH descriptor: [Coronaviridae] explode all trees
4	MeSH descriptor: [Betacoronavirus] explode all trees
5	coronavir* OR corona virus OR betacoronavir* OR beta coronavirus
6	MeSH descriptor: [COVID-19] explode all trees
7	COVID19 OR COVID 19 OR COVID 2019
8	nCoV OR CoV 2 OR CoV2 OR 2019 nCoV
9	MeSH descriptor: [SARS-CoV-2] explode all trees
10	severe acute respiratory syndrome coronavirus 2 OR SARS CoV 2 OR sarscov2
11	#1 OR #2 OR #3 OR #4 OR #5 OR #6 OR #7 OR #8 OR #9 OR #10
12	MeSH descriptor: [Vaccines] explode all trees
13	MeSH descriptor: [immunity] explode all trees
14	MeSH descriptor: [Immunogenicity, Vaccine] explode all trees
15	(vaccin*):ti,ab,kw OR (immuniz*):ti,ab,kw OR (inocul*):ti,ab,kw
16	#12 OR #13 OR #14 OR #15
17	#11 AND #16 (1413)
18	MeSH descriptor: [COVID-19 Vaccines] explode all trees
19	COVID19 Vaccin* OR COVID 19 Vaccin* OR anti sars cov 2 agent OR anti sarscov2 agent OR sars cov 2 vaccin* OR sarscov2 vaccin*
20	#18 OR #19
21	#17 OR #20 (1498)
22	(boost*):ti,ab,kw OR (third):ti,ab,kw
23	#21 AND #22 (259)
24	(human*):ti,ab,kw OR (volunteer*):ti,ab,kw OR (participant*):ti,ab,kw OR (subject *):ti,ab,kw OR (people*):ti,ab,kw
25	#23 AND #24 (201)
26	trial: 191

Table S3. Literature search terms used for Embass. The final search strategy applied to conduct this meta-analysis is reported at step #20.

#	**Search strategy**
1	coronavirus OR "corona virus"
2	'coronavirinae'/exp OR coronavirinae
3	'coronaviridae'/exp OR coronaviridae
4	'betacoronavirus'/exp OR betacoronavirus OR 'beta coronavirus'
5	'coronavirus disease 2019'/exp OR 'coronavirus disease 2019' OR 'covid-19' OR covid19 OR sarscov2 OR 'covid-2019' OR covid2019
6	'severe acute respiratory syndrome coronavirus 2'/exp OR 'severe acute respiratory syndrome coronavirus 2' OR 'ncov' OR 'sars-cov-2'
7	'cov 2' OR cov2
8	'2019-ncov' OR 2019ncov
9	#1 OR #2 OR #3 OR #4 OR #5 OR #6 OR #7 OR #8
10	'vaccine'/exp OR vaccin*:ti,ab,kw
11	'immunization'/exp OR immuniz*:ti,ab,kw
12	inocul*:ti,ab,kw
13	#10 OR #11 OR #12
14	#9 AND #13 (4631)
15	'anti-sars-cov-2 agent'/exp OR 'anti-sars-cov-2 agent' OR 'sars-cov-2 vaccine'/exp OR 'sars-cov-2 vaccine' OR "covid19 vaccin*" OR "covid 19 vaccin*"
16	#14 OR #15 (5054)
17	boost*:ti,ab,kw OR third:ti,ab,kw
18	#16 AND #17 (585)
19	'human'/exp OR human:ti,ab,kw OR volunteer*:ti,ab,kw OR participant*:ti,ab,kw OR subject*:ti,ab,kw OR people:ti,ab,kw
20	#18 AND #19 (539)

Table S4. Literature search terms used for Medrxiv, Wanfang and CNKI.

**Database**	**Search strategy**
Medrxiv	COVID-19 AND vaccin* AND (boost* OR third) (734)
Wanfang	(主题:(新型冠状病毒) or 主题:(新冠病毒) or 主题:(新冠) or 主题:(COVID-19) or 主题:(SARS-CoV-2)) and 主题:(疫苗) and (主题:(加强针) or 主题:(加强免疫) or 主题:(第三针)) (68)
CNKI	TKA=('新型冠状病毒'+'新冠病毒'+'新冠'+'新冠肺炎'+'COVID-19'+'SARS-CoV-2') and TKA=('疫苗') and TKA=('加强针'+'加强免疫'+'第三针') (64)

"主题" means "Theme"; "新型冠状病毒,新冠病毒,新冠,新冠肺炎" mean "COVID-19"; "疫苗" means "Vaccine"; "加强针" means "Booster Dose"; "加强免疫" means "Boost Immunity"; "第三针" means "Third Dose"

Table S5. Results of Begg's test in different outcomes.

Outcome	Vaccination regimen	Number of included studies	Begg's test	
			Z	*P* value
Log-transformed neutralisation antibody titres	homologous	16	-0.63	0.53
Log-transformed anti-RBD IgG	homologous	8	-0.74	0.46
Seropositive rate of antibodies	homologous	18	4.02	**<0.01**
Log-transformed neutralisation antibody titres	heterologous	9	-1.67	0.10
Log-transformed anti-RBD IgG	heterologous	11	-1.32	0.19
Seropositive rate of antibodies	heterologous	5	2.45	**0.02**
Total adverse events	homologous	10	1.44	0.15
Total adverse events	heterologous	7	0.30	0.76

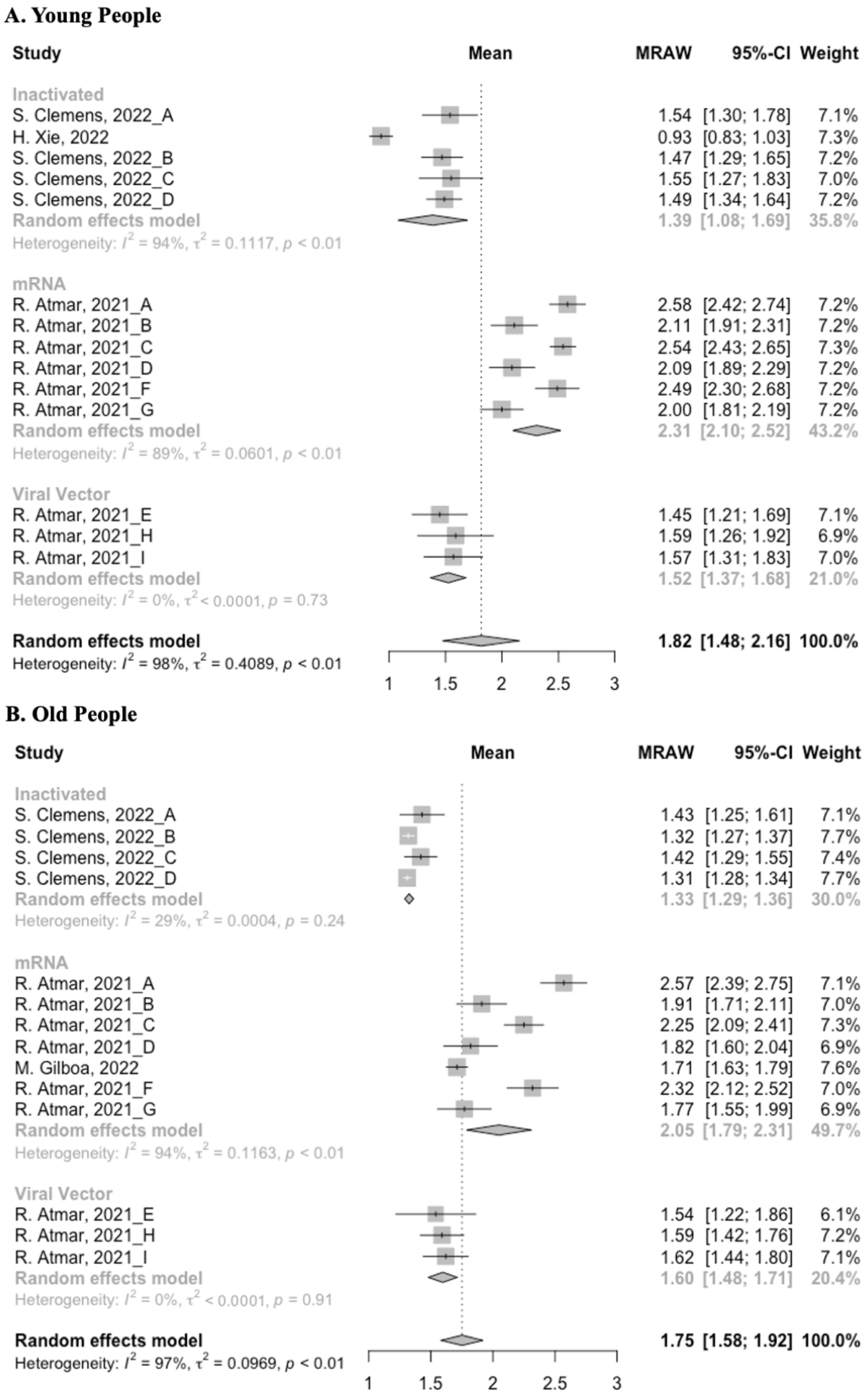

Figure S1. Forest plot of the pooled log-transformed neutralisation antibody titres before booster vaccination in different age groups

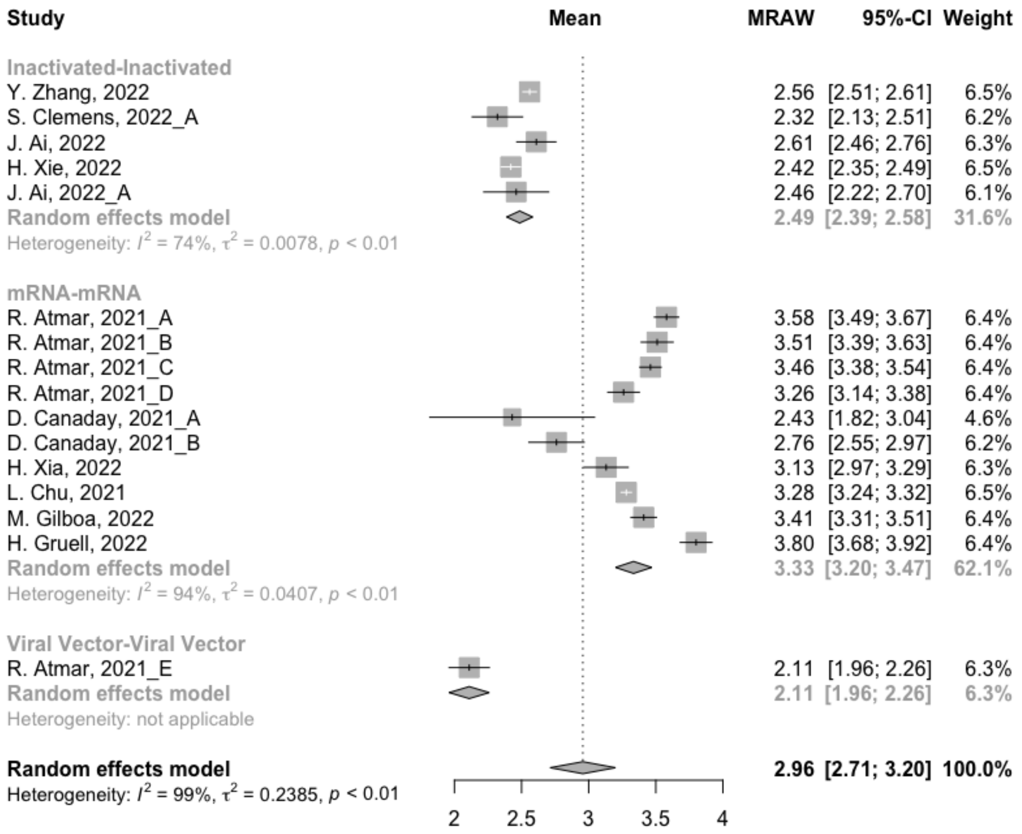

Figure S2. Forest plot of the pooled log-transformed neutralisation antibody titres after homologous booster vaccination

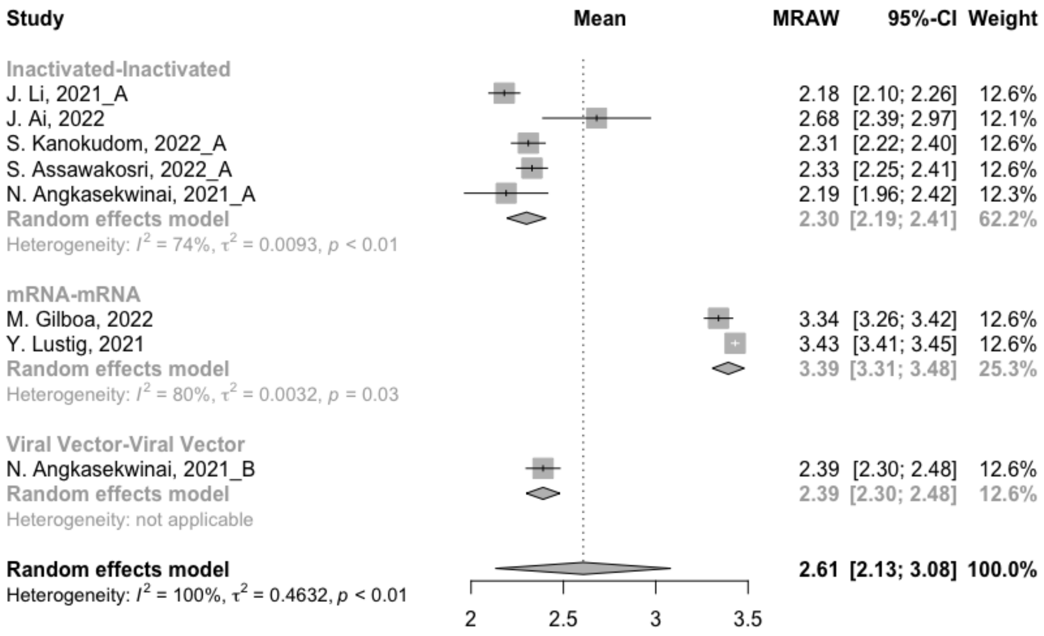

Figure S3. Forest plot of the pooled log-transformed anti-RBD IgG after homologous booster vaccination

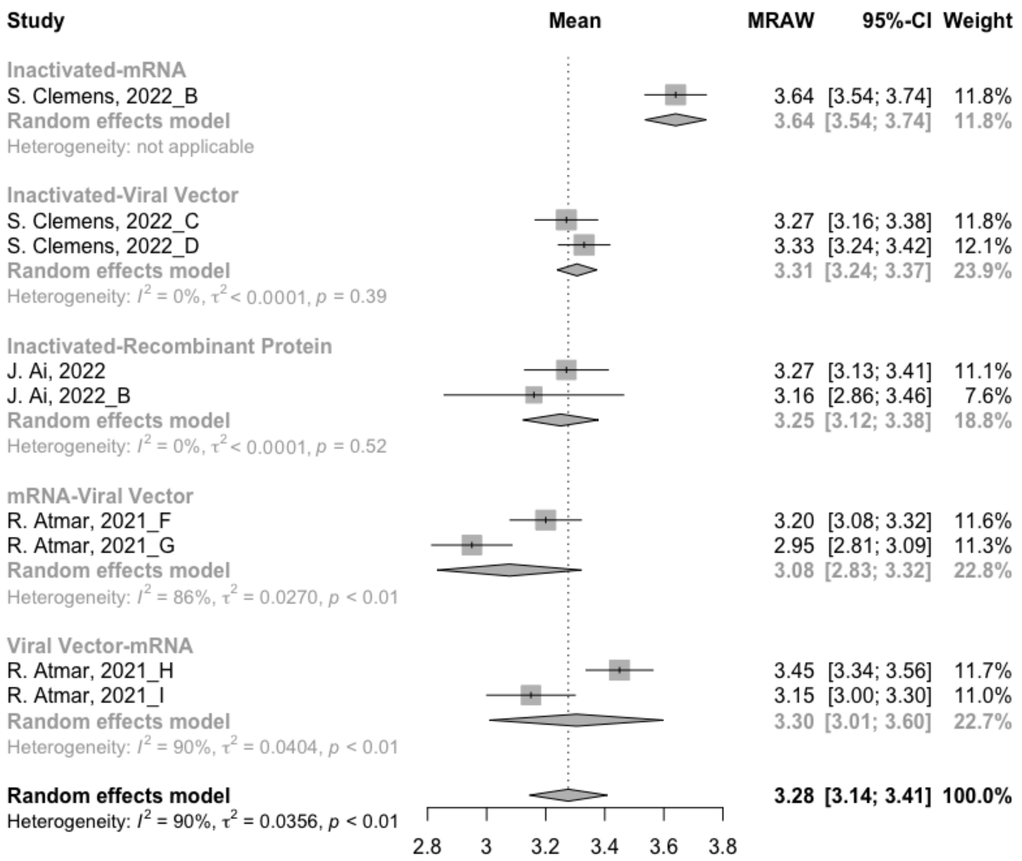

Figure S4. Forest plot of the pooled log-transformed neutralisation antibody titres after heterologous booster vaccination

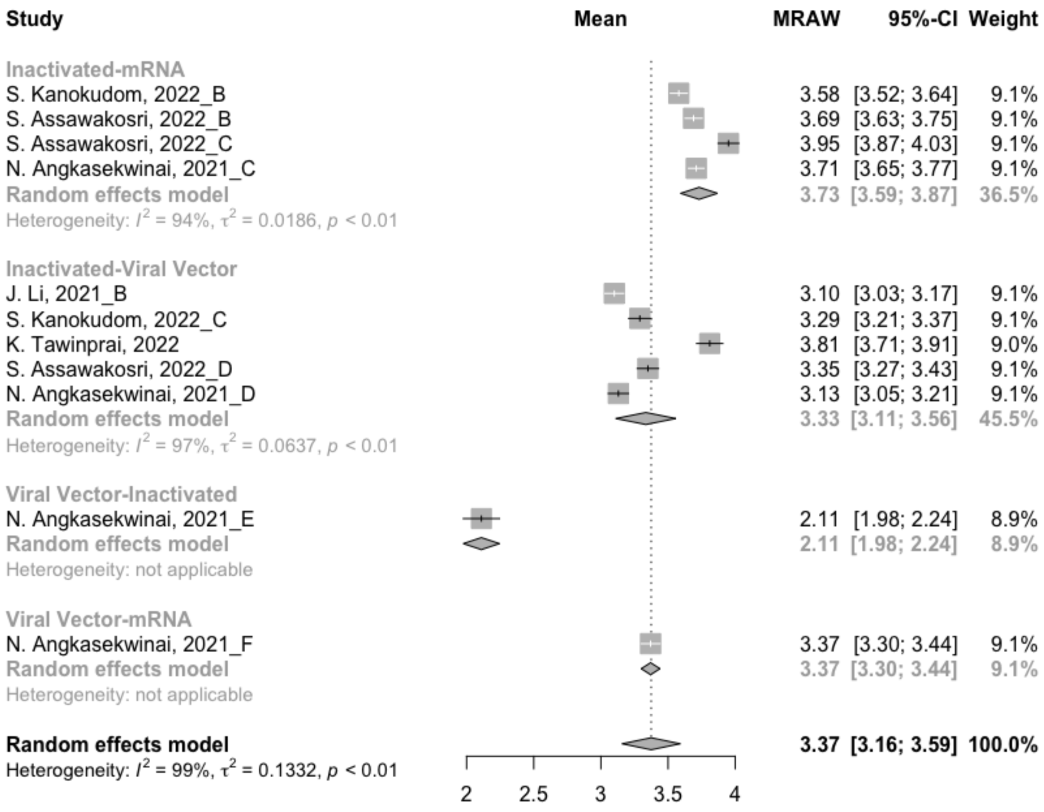

Figure S5. Forest plot of the pooled log-transformed anti-RBD IgG after heterologous booster vaccination

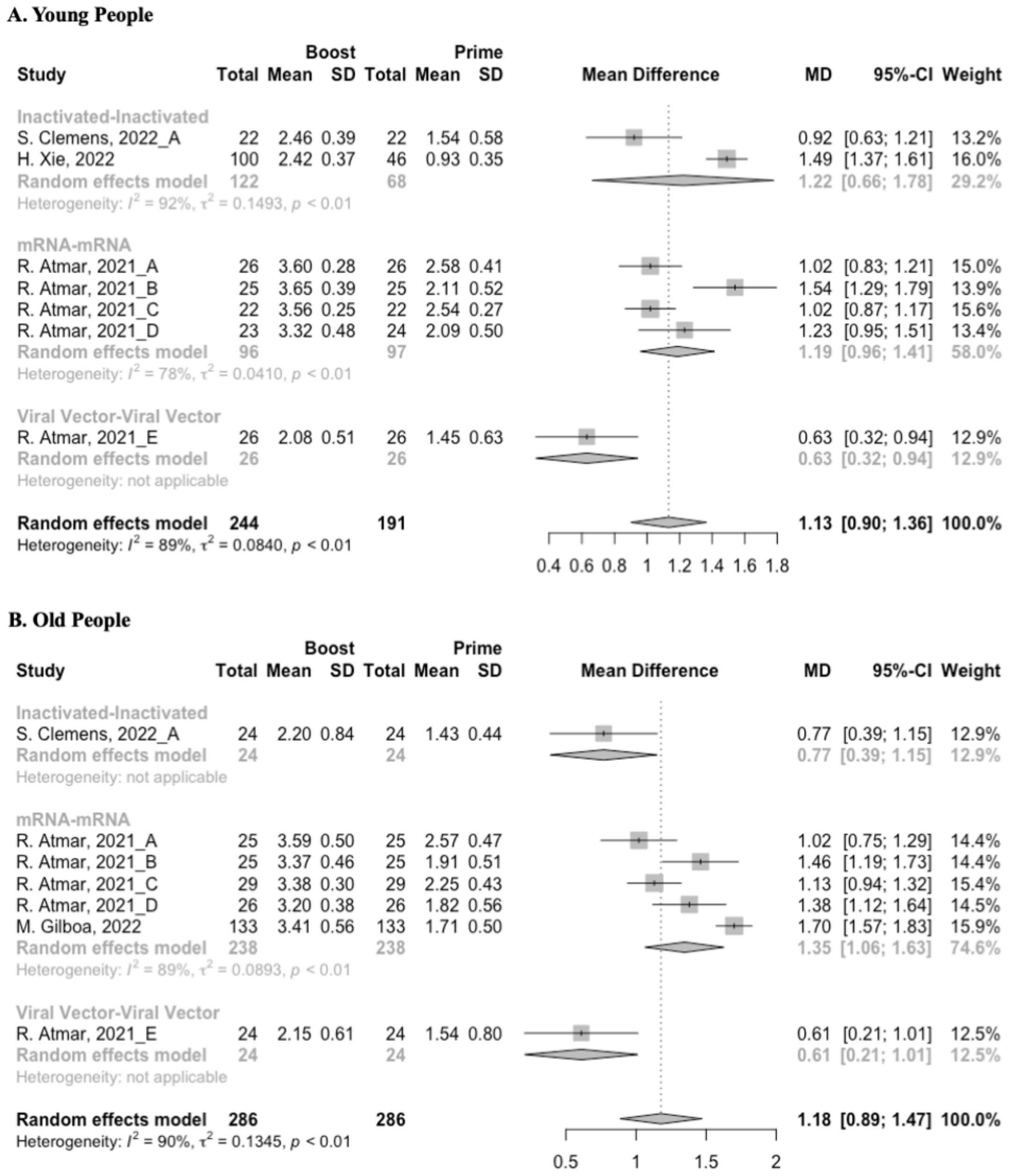

Figure S6. Forest plot of the pooled log-transformed neutralisation antibody titres between before and after homologous booster vaccination in different age groups

A. Young People

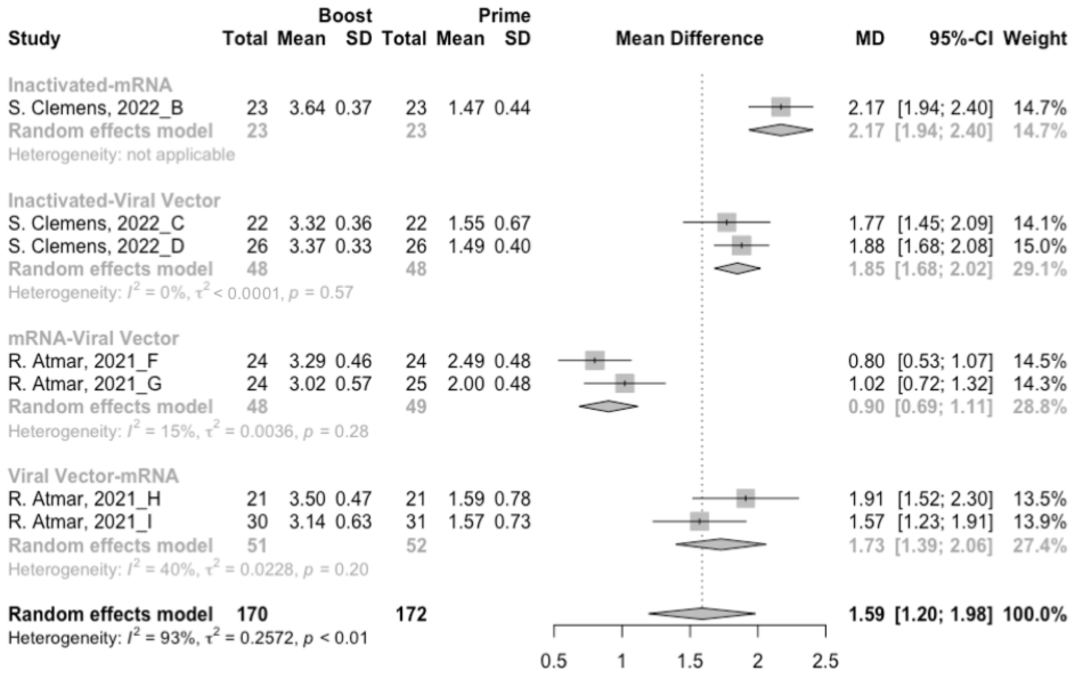

B. Old People

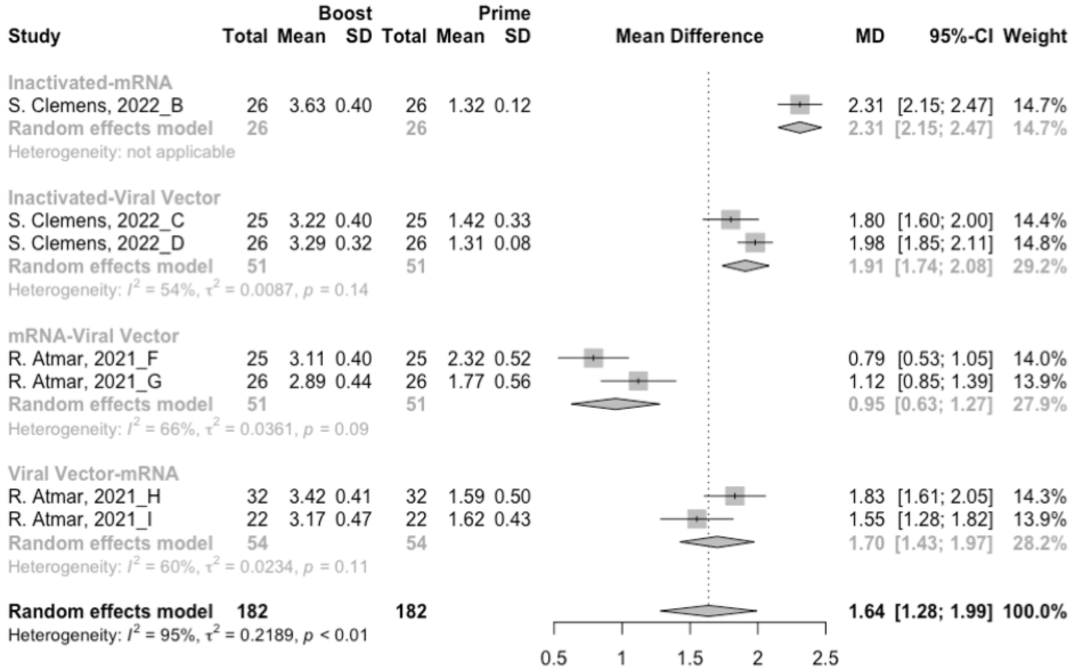

Figure S7. Forest plot of the pooled log-transformed neutralisation antibody titres between before and after heterologous booster vaccination in different age groups
